# Advancements and Strategies in Robotic Planning for Knee Arthroplasty in Patients with Minor Deformities

**DOI:** 10.3390/life14121528

**Published:** 2024-11-21

**Authors:** Giacomo Capece, Luca Andriollo, Rudy Sangaletti, Roberta Righini, Francesco Benazzo, Stefano Marco Paolo Rossi

**Affiliations:** 1 Sezione di Chirurgia Protesica ad Indirizzo Robotico—Unità di Traumatologia dello Sport, Ortopedia e Traumatologia, Fondazione Poliambulanza, 25124 Brescia, Italy; 2 Ortopedia e Traumatologia, Università Cattolica del Sacro Cuore, 00168 Rome, Italy; 3 Artificial Intelligence Center, Alma Mater Europaea University, 1010 Vienna, Austria; 4 Biomedical Sciences Area, IUSS Istituto Universitario di Studi Superiori, 27100 Pavia, Italy

**Keywords:** robotic-assisted surgery, knee arthroplasty, Persona knee system, preoperative planning, surgical outcomes

## Abstract

Knee arthroplasty, commonly performed to treat osteoarthritis, necessitates precise surgical techniques for optimal outcomes. The introduction of systems such as the Persona Knee System (Zimmer Biomet, Warsaw, IN, USA) has revolutionized knee arthroplasty, promising enhanced precision and better patient outcomes. This study investigates the application of robotic planning specifically in knee prosthetic surgeries, with a focus on Persona Knee System prostheses. We conducted a retrospective analysis of 300 patients who underwent knee arthroplasty using the Persona Knee System between January 2020 and November 2023, including demographic data, surgical parameters, and preoperative imaging. Robotic planning was employed to simulate surgical procedures. The planning process integrated preoperative imaging data from a specific program adopted for conducting digital preoperative planning, and statistical analyses were conducted to assess correlations between patient characteristics and surgical outcomes. Out of 300 patients, 85% presented with minor deformities, validating the feasibility of robotic planning. Robotic planning demonstrated precise prediction of optimal arthroplasty sizes and alignment, closely aligning with preoperative imaging data. This study highlights the potential benefits of robotic planning in knee arthroplasty surgeries, particularly in cases with minor deformities. By leveraging preoperative imaging data and integrating advanced robotic technologies, surgeons can improve precision and efficacy in knee arthroplasty. Moreover, robotic technology allows for a reduced level of constraint in the intraoperative choice between Posterior-Stabilized and Constrained Posterior-Stabilized liners compared with an imageless navigated procedure.

## 1. Introduction

Total knee arthroplasty (TKA) is a critical procedure for treating conditions such as osteoarthritis (OA), rheumatoid arthritis, and post-traumatic arthritis [1]. It involves the replacement of damaged or diseased knee joints with prosthetic components in order to alleviate pain, improve mobility, and enhance overall quality of life for affected individuals [2,3]. With the global population aging and the prevalence of degenerative joint diseases increasing, the demand for knee arthroplasty procedures is expected to rise significantly in the coming years [4].

When thinking about knee arthroplasty, the inherent variability in human anatomy and the complexity of knee joint biomechanics pose significant challenges. Moreover, the success of knee replacement surgery heavily depends on precise alignment, proper sizing of arthroplasty components, and optimal soft tissue balancing to ensure the stability and function of the freshly operated joint [5]. Additionally, in cases of severe arthritis or previous fractures, further surgical precision is required [6].

Robotic-assisted surgical technologies have been shown to enhance surgical precision, accuracy, and reproducibility in orthopedic procedures [7]. Equipped with advanced imaging modalities and sophisticated software algorithms, robotic systems enable surgeons to meticulously plan and execute surgical procedures with unmatched control and fidelity. For instance, robotic-assisted surgeries have been shown to significantly reduce surgical errors compared with traditional methods. Furthermore, the precision of robotic systems contributes to a decrease in postoperative complications, underscoring the significant impact of these technologies on patient outcomes [8]. By providing real-time feedback and intraoperative guidance, robotic platforms empower surgeons to overcome the limitations of manual techniques, thereby raising the standard of care in knee arthroplasty [9]. This technological advancement aligns with recent trends in knee arthroplasty, where there has been a consistent increase in both the quantity and quality of implants. Individual anatomical variability has been emphasized, leading to the development of various implant types characterized by differences in cuts, patellar design, or tibial tray type. This has led to the development of custom-made prostheses, which differ from standard options by being specifically designed to fit the unique anatomy of each patient, offering benefits such as enhanced fit and comfort, improved functionality, reduced surgical time, and personalized care.

The Persona Knee System (Zimmer Biomet, Warsaw, IN, USA) represents a cutting-edge advancement in knee arthroplasty technology, designed to optimize implant fit, stability, and longevity while minimizing the risk of complications and implant failure [10,11]. These prostheses incorporate innovative features such as personalized sizing options, anatomically shaped components, and advanced materials to closely mimic the natural kinematics of the knee joint. The anatomical shape of the tibial component in the Persona Knee System (Zimmer Biomet, Warsaw, IN, USA) may result in a better fit and less overhang of the tibial tray. Moreover, the availability of large component sizes with 1 mm thickness increments of the inlays could potentially improve knee stability post-surgery [12,13]. This feature, along with other advanced characteristics, represents a significant strength of the Persona Knee System (Zimmer Biomet, Warsaw, IN, USA). While this modification is indeed common among contemporary total knee arthroplasty systems, the specific design and customization options offered by the Persona Knee System enhance its adaptability to individual patient needs and overall patient outcomes. Consequently, they provide an ideal platform for assessing the effectiveness of robotic planning in knee arthroplasty surgeries, particularly in cases involving minor deformities where precise alignment and sizing are crucial. The advancements in knee implants, the introduction of new alignment techniques, and the opportunities afforded by robotics have increasingly emphasized the need for preoperative planning in arthroplasty surgery. Nowadays, several digital preoperative planning programs have emerged, such as robotic image-based or imageless planning and custom-made implants with prior CT planning. Preoperative planning involves preparing for surgery by assessing the patient’s condition and determining the optimal surgical approach to achieve the best outcomes. Digital preoperative planning programs enhance this process with advanced technologies. Robotic image-based planning uses 3D models generated from CT scans to provide precise guidance during surgery. Imageless planning employs algorithms and data from physical exams or general anatomical information, bypassing specific imaging and relying on computational methods to strategize the procedure.

An example is the Sectra system (Sectra AB, Teknikringen, Linköping, Sweden), produced by a medical imaging solutions company who have introduced a new osteotomy tool in their radiographic system [14]. This underscores the importance of integrating technological advancements into surgical practice to optimize patient outcomes.

The Rosa (Robotic Surgical Assistant) Knee System (Zimmer Biomet, Warsaw, IN, USA) aids the surgeon in performing the distal femoral cut, sizing and positioning the femoral component, making the tibial cut, and achieving ligament balance. This robotic system provides two options: image-based or image-free. Specifically, the image-based option uses 2D X-rays that are transformed into a digital 3D replication of the patient’s anatomy, while the image-free system relies entirely on the acquisition of intraoperative landmarks. Various surgical techniques can be employed, including measured resection, gap balancing, functional alignment, and kinematic alignment [15]. In our institution, we utilize the imageless mode, which allows for real-time adjustments during surgery by referencing anatomical landmarks. This approach enhances precision and adaptability, ensuring that implant sizing and positioning are tailored to the individual patient’s anatomy.

The primary aim of this study was to investigate the application and efficacy of robotic planning in knee replacement surgeries involving the Persona Knee System (Zimmer Biomet, Warsaw, IN, USA), particularly in cases with minor deformities. The goal was to determine whether specific sizes of prosthetic components are more frequently used, thus establishing standardized measurements for patients with minor deformities.

The secondary objective of this study was to evaluate whether the robotic technique allows for the intraoperative choice of a reduced constraint between Posterior-Stabilized and Constrained Posterior-Stabilized liners, compared to an imageless navigated procedure.

## 2. Methods

### 2.1. Study Design and Inclusion Criteria

This study involved a retrospective analysis of data from 300 patients who underwent knee arthroplasty procedures using Persona Knee System (Zimmer Biomet, Warsaw, IN, USA) prostheses between January 2020 and November 2023 at the robotic arthroplasty surgery unit of a single high-volume center specializing in knee arthroplasty surgery. Enrolled patients underwent TKA surgery with the Persona Knee System assisted by the Rosa Knee System robot (Zimmer Biomet, Warsaw, IN, USA).

Patients included in the study had minor knee deformities and showed a maximum 15 degrees varus or 10 degrees valgus deviation [5,16]. The selection of patients from this timeframe ensured a contemporary representation of surgical practices and outcomes, thereby enhancing the relevance and applicability of the study findings to current clinical practice. No patients were excluded from this analysis as they were retrospectively selected based on the inclusion criteria of our study, ensuring a comprehensive overview of outcomes with this specific prosthesis type.

For the comparison with patients treated with an imageless navigated procedure, 141 patients treated with iAssist (Zimmer Biomet, Warsaw, IN, USA) were enrolled between October 2019 and April 2020 in the same unit specializing in knee arthroplasty surgery. With this navigated technique, the same type of prosthesis, the Persona Knee System (Zimmer Biomet, Warsaw, IN, USA), was implanted.

The two compared groups [300 patients for robotic-assisted TKA (r-TKA) and 141 for iAssist] have different sample sizes as they represent the actual cases from a single center from January 2020 to November 2023. All patients analyzed and undergoing surgery were operated on by the same surgeons belonging to the same unit, who are experienced in robotic and navigated prosthetic surgery.

### 2.2. Data Collection

Demographic data, including age and gender, were documented to characterize the study cohort comprehensively. Additionally, relevant surgical parameters such as femoral and tibial component sizes were recorded to elucidate the anatomical characteristics of the patient population and their implications for surgical planning and outcomes. Specifically, we analyzed the use of Posterior-Stabilized (PS) or Cruciate-Retaining (CR) prostheses. PS prostheses are designed to replace the function of the posterior cruciate ligament (PCL) with a mechanical cam and post mechanism, while CR prostheses are designed to preserve the patient’s natural PCL for more natural knee movement. We also analyzed standard or narrow implants, the use of Stem Extension or the absence thereof, insert dimensions, and whether the type of insert was PS, Medial Congruent (MC), or Constrained Posterior-Stabilized (CPS). The choice of PS or CR prostheses, and the use of standard or narrow implants, were analyzed to understand their impact on alignment and stability.

### 2.3. Type of Liner and Alignment Used

The study was conducted at a single center, with patients treated by three different surgeons. The choice of femoral implant type was primarily based on the surgeon’s preference. However, an increase in the use of CR femoral components with MC liners was observed. The MC liner has been introduced as an evolution of the ultracongruent (UC) liner. The design of the MC provides medial anteroposterior stability through the presence of the anteroposterior lip, while the flat surface of the lateral inlay allows for greater freedom of movement of the respective condyle. Both inlays can be implanted using the same CR femur, preserving the intercondylar bone and reducing the risk of intraoperative fractures. The MC inlay allows for both PCL-retaining and PCL-sacrificing, whereas the UC only allows for PCL-sacrificing [17].

The benefits of using the MC insert appear promising. However, in cases of preoperative ligament instability or the risk of intraoperative ligament instability, it is advisable to opt for a PS implant. The PS implant allows for the use of an increased constraint insert, the CPS. A difference of more than 3 mm (ranging from 3 to 5 mm) between the medial and lateral compartments at 0° and 30° following the distal femoral and proximal tibial cuts, or medial laxity greater than 3 mm at 60° or 90°, indicated the necessity for a CPS liner.

The alignment used was patient-specific, according to the study published by Rossi and Benazzo, where it was defined as Individualized Alignment [18]. This type of alignment is essentially an inverse restricted kinematic, fitting within the more modern philosophy of Functional Alignment [19].

### 2.4. Robotic Planning and Integration of Preoperative Imaging Data

All enrolled and analyzed patients underwent knee radiographs in anteroposterior, lateral, axial patellar, Rosenberg projections, and a lower-limb weight-bearing radiograph. The anteroposterior projection provides a clear view of the joint space and alignment of the knee, while the lateral projection offers insight into the patellar position and any potential posterior knee abnormalities. The axial patellar projection is crucial for assessing the patellofemoral joint and detecting patellar maltracking. The Rosenberg projection, taken with the knee in a flexed position, is particularly valuable for identifying early degenerative changes in the posterior tibial plateau. Lastly, the lower-limb weight-bearing radiograph evaluates overall limb alignment and the mechanical axis, which are critical for planning surgical interventions and ensuring proper prosthetic placement. Digital preoperative planning was conducted using Sectra based on the latter projection. Robotic planning software was used to precisely manipulate arthroplasty components and provide real-time visualization of intraoperative variables, allowing for customized treatment strategies, empowering surgeons to customize treatment strategies according to individual patient anatomy and pathology.

At the core of successful robotic planning was the integration of imaging data obtained from Sectra, a sophisticated imaging platform known for its high-resolution imaging capabilities and comprehensive anatomical visualization. Surgeons were able to obtain detailed insights into the patient’s knee anatomy, including bone morphology and pathological changes.

This comprehensive preoperative assessment facilitated accurate previsualization of the surgical site and informed the selection of appropriate implant sizes and configurations.

### 2.5. Impact of Robotic Technique on the Choice of Insert Type

The impact of robotic techniques compared to iAssist (Zimmer Biomet, Warsaw, IN, USA), an imageless navigated procedure, was also evaluated. Demographic characteristics were compared, as well as the type of insert used in terms of constraint (PS vs. CPS) and thickness. To enable evaluation of the intraoperative choice, patients who had a CR femoral implant with an MC insert were excluded, as this choice was made preoperatively.

### 2.6. Statistical Analysis

A power analysis was conducted using the G*Power sample size calculator. For a one-tailed Mann–Whitney U test with a medium effect size (0.5), a significance level of 0.05, and a desired power of 0.80, the required sample size is approximately 51 participants per group. For a one-tailed chi-square test with a medium effect size (0.3), a significance level of 0.05, and a desired power of 0.80, the required total sample size is approximately 87 participants. The sample enrolled in this study achieves adequate statistical power for the analysis.

Statistical analysis was performed using Excel software (version 2016; Microsoft, Redmond, WA, USA) by an independent statistician. Excel was chosen for its accessibility and familiarity, which facilitated the efficient handling of data and execution of basic statistical functions. Excel’s capabilities were deemed sufficient for the scope of this analysis.

Continuous variables were reported using averages and standard deviations (SD) and categorical variables were presented using frequency distributions and percentages.

For categorical variables, the chi-squared test was employed to assess differences in proportions between the two groups. For continuous variables, the Mann–Whitney U test was used to compare the medians between groups.

Statistical significance was maintained at a *p* value of less than 0.05.

The Level of Evidence for this study is III, as it is a retrospective cohort study.

## 3. Results

### 3.1. Demographics

The patient cohort consisted of 110 males (36.7%) and 190 females (63.3%), with ages ranging from 38 to 92 years (mean 72.7 ± 8.9 years). Surgical procedures were evenly distributed between the right (150 patients) and left (150 patients) sides. The demographic data are summarized in Table 1. All patients undergoing surgery were afflicted with primary, post-traumatic secondary, or rheumatologic-related knee OA, with a maximum varus deviation of 15 degrees or a valgus deviation of 10 degrees.

### 3.2. Prosthetic Types and Result Heterogeneity

The sample’s heterogeneity was evident in the choice of prosthetic types. PS prostheses were predominantly used in 268 patients (89.3%), while CR prostheses were used in 32 patients (10.6%). Regarding the femoral components utilized in a diverse sample of 300 individuals over the past three years in one center, size 3 was used only once (0.3%), size 4 was used 8 times (2.7%), size 5 was used 37 times (12.3%), size 6 was used 49 times (16.3%), size 7 was used 53 times (17.7%), size 8 was used 47 times (15.7%), size 9 was used 48 times (16%), size 10 was used 25 times (8.3%), size 11 was used 27 times (9%), and size 12 was used 5 times (1.6%). Table 2 presents the data on the types of femoral components.

**Figure 1:**
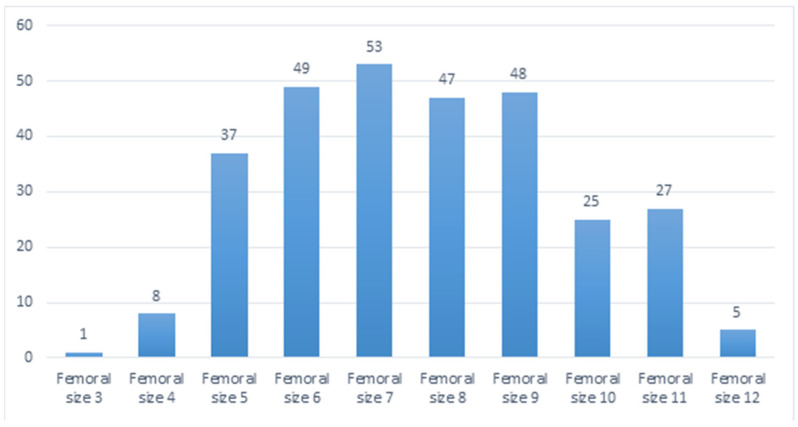
Distribution of femoral components.

Figure 1 shows the graph of the distribution of femoral components. The statistical analysis went further, examining the specific type of femoral component adopted and whether it be standard or narrow. Specifically, 57 standard femoral prostheses (19%) and 243 narrow femoral prostheses (81%) were employed.

**Figure 2:**
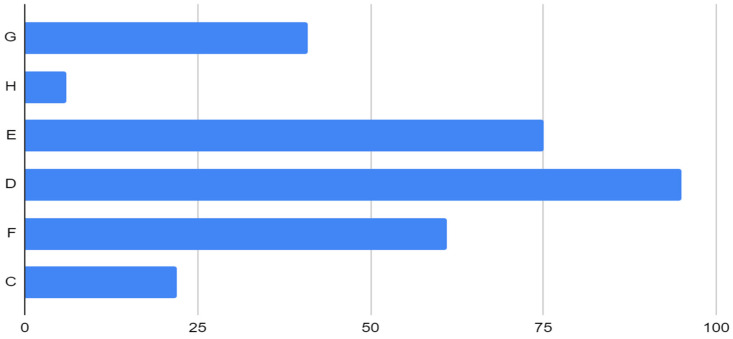
Distribution of tibial components.

The analysis highlighted that tibial sizes D, E, and G were predominantly used (57%). Table 3 presents the data on the types of tibial components. Tibial components were predominantly size D (31.7%) and E (25%), highlighting a preference for these sizes in the study population. Figure 2 shows the graph of the distribution of femoral components. The Stem Extension was adopted in a total of 259 patients (86.3%).

In the 300 enrolled patients, the predominant use of the 10 mm polyethylene (PE) insert was observed, accounting for 64.7%, followed by the 11 mm insert at 23% and the 12 mm insert at 12.3%. PS inserts were the most commonly used type, comprising 71.3% of cases. A MC insert was used in 53 cases (17.7%), while a CPS insert was used in 33 cases (11%). Table 4 presents the data on the types of inserts used and their thickness.

Subsequently, after this descriptive analysis of the treatment process with arthroplasty that involves preoperative planning and intraoperative use of robotic techniques, a comparison was made between the use of robotic techniques and non-robotic surgical techniques in the impact they can have on the choice of constraint between PS and CPS.

Specifically, the results of 141 patients treated with iAssist (Zimmer Biomet, Warsaw, IN, USA) were analyzed. No statistically significant differences emerged, either in terms of age (*p* value = 0.197) or gender (*p* value = 0.372). The complete data from this analysis are reported in Table 5.

After excluding statistically significant differences between the two groups, the percentages of PS and CPS liner usage, as well as the relative height of the inserts, were compared. A statistically significant difference emerged in the use of the increased constraint CPS insert, which was greater in the group treated without robotic surgery (*p* value = 0.013). The complete data from the statistical analysis are summarized in Table 6.

As for the average surgical time, it was found to be almost comparable between the robot-assisted procedure and the navigated one. It goes without saying that, since these procedures were performed by different experienced surgeons, the average time may vary among the different surgeons but is comparable when comparing the two different surgical procedures.

## 4. Discussion

### 4.1. Technological Advancements in TKA

TKA is a common orthopedic procedure aimed at relieving the severe symptoms of OA and other degenerative joint diseases. As shown in this study, it is often performed in older adult patients. In recent years, the landscape of knee replacement surgery has undergone a paradigm shift; the conventional operating technique has become outdated with the introduction of imageless robotic-assisted surgical technologies [15]. Robotic systems provide real-time feedback and intraoperative guidance, resulting in more precise bone cuts and optimal implant placement [20,21]. The utilization of Personalized Alignment techniques relies on implant customization to accommodate the wide spectrum of human knee anatomies. Unlike conventional one-size-fits-all approaches, personalized implant orientation, particularly with kinematic alignment (KA), mitigates bone–implant mismatches, thereby enhancing prosthetic fit and alignment accuracy. The advent of a custom TKA system exemplifies this shift towards anatomy-based customization, aiming to replicate the native knee anatomy pre-arthritically [22].

Custom-made implants in TKA significantly enhance clinical outcomes and patient satisfaction by offering a tailored fit that matches the unique anatomy of each patient. This personalized approach leads to improved joint function, reduced postoperative pain and a shorter recovery time. Additionally, custom implants increase overall patient satisfaction by addressing individual needs and optimizing surgical precision. Through meticulous pre-manufacturing planning, engineers can develop a roadmap, ensuring traceability and precision in implant fabrication. Consequently, personalized implants mitigate risks of prosthetic overhang or under-coverage [23].

Having all components available requires rigorous organizational management for inventory and warehousing. This is particularly pertinent in the field of healthcare, where the timely availability of medical implants and equipment directly impacts patient care and outcomes. Above all, smaller healthcare facilities often face challenges in maintaining adequate inventories due to limited resources and storage space. In such settings, implementing strategies for inventory optimization becomes imperative to ensure the availability of essential medical supplies while minimizing wastage and costs.

### 4.2. Clinical Implications of Robotic-Assisted Surgery

This study contributes to this evolving field by investigating the application of robotic planning specifically in knee arthroplasty surgeries with a particular focus on the utilization of Persona Knee System prostheses. Personalized Alignment represents an advancement from traditional TKA, leveraging extensive robotic assistance to precisely replicate patient anatomy, account for individual soft tissue characteristics, and ensure precise placement of components. By prioritizing personalized adjustments tailored to each patient’s unique physiology, Personalized Alignment strives to achieve superior results in clinical outcomes compared with conventional methods, ultimately contributing to improved overall patient satisfaction and well-being [24].

The primary objective of this research was to evaluate the effectiveness of robotic planning in cases presenting with minor deformities and to assess its compatibility with preoperative imaging modalities such as Sectra.

According to Batailler et al. [25], rTKA offers several benefits, including precise alignment of the knee, accurate positioning of implants, optimal ligament balance based on real-time bone cutting, and protection of soft tissues. This approach enhances surgical precision, potentially leading to improved postoperative outcomes such as better joint stability, reduced risk of complications, and enhanced overall function and comfort for patients, enhancing patient-reported satisfaction outcomes [26,27].

Sparmann et al. identified a statistically significant variance in component positioning and overall limb alignment, while Mancino et al. reported that robotic-assisted knee arthroplasty yields superior short-term clinical outcomes, characterized by a high rate of patient satisfaction [28,29]. However, they both noted no remarkable disparities in survivorship or complication rates when compared to conventional manual surgery.

### 4.3. Study Findings

Furthermore, referring to the statistical analysis we identified a correlation between patient age and femoral size. It seems that younger patients often require larger arthroplasty components, both femoral and tibial sizes. This highlights the importance of customizing implant selection to accommodate age-related variations in knee anatomy. Studies show that anatomical differences in knee joints become more pronounced with age, affecting the fit and function of standard implants. For instance, research indicates that approximately 30% of patients over the age of 65 have anatomical variations that are not adequately addressed by conventional implants. This can lead to increased rates of misalignment and dissatisfaction. Similarly, gender-based differences were observed in tibial size, with males exhibiting a tendency towards larger components compared with females. These gender-specific differences underscore the necessity of tailored surgical planning based on patient sex, as variations in knee anatomy between genders can significantly impact implant selection and surgical outcomes. Additionally, ethnic distribution must be considered, as ethnic differences are pivotal in the size distribution of femoral and tibial components. Kim et al. highlight that morphologic features of the knee vary among patients of different races, emphasizing the importance of incorporating ethnic diversity into surgical planning to optimize implant fit and patient outcomes [30].

Robotic planning has demonstrated a precise prediction of optimal arthroplasty sizes and alignment, closely adjusting with preoperative imaging data obtained from Sectra [31]. Notably, it allows for the balancing of flexion space without the need for personalized planning, offering a significant advantage in streamlining surgical workflows and enhancing surgical efficiency. Sometimes, balancing the flexion space may require down/up sizing of the implant, if compatible with the medio-lateral dimensions of the condyle. This adjustment allows for 2 mm of play without altering the extension space.

Finally, this study examined two distinct arthroplasty types: PS and CR variants. Following these findings it was observed that a significant majority, accounting for 95% of patients undergoing rTKA, received the PS prosthetic implantation. As indicated by Sun et al. and Kulshrestha et al., at the initial follow-up, a notable disparity in range of motion (ROM) was observed between the PS and CR knee implants, while no substantial variance was detected in other comparisons [32,33]. However, upon long-term follow-up, no statistically significant rise in ROM was noted in any particular knee implant relative to the others, just as there were no disparities regarding complications and revisions associated with implant safety. Further research is warranted to validate these findings and explore the long-term implications of robotic-assisted techniques in orthopedic surgery. As the field continues to evolve, continued advancements in robotic-assisted technologies hold promise for further improving surgical outcomes and addressing the evolving needs of patients with degenerative joint diseases.

Compared with navigated techniques, robot-assisted surgery significantly reduces variability during the procedure, particularly in selecting and placing implants. Although navigation is recognized as a useful tool, it heavily depends on the surgeon’s manual skills, which can introduce greater variability based on the operator. On the other hand, robotic systems offer a higher level of control and precision, reducing the need for adjustments during the operation and enhancing overall surgical outcomes [34,35].

The increased precision reported in the literature is due to the surgeon’s ability to use dedicated software for intraoperative planning to determine the optimal thickness and angle of bone resection, aiming to achieve a well-aligned and balanced TKA. The robotic technique allows for real-time assessment and adjustment of resection thickness, joint gaps, and limb alignment during the surgical procedure. The surgeon thus has precise data with which to evaluate the joint during the various phases of replacement. In detail, as reported in a study by Rossi et al., a discrepancy greater than 3 mm (between 3 and 5 mm) between the medial and lateral compartments at 0° and 30° after the distal femoral and proximal tibial cuts, or medial laxity at 60° or 90° of more than 3 mm, indicated the need for a CPS implant [5].

This precision and the ability to evaluate at various steps of the surgical procedure may explain the statistically significant difference found in this study in the use of reduced constraint through robotic techniques. Indeed, with this tool, it is possible to select the degree of constraint more accurately and objectively, without having to rely on the manual experience of the individual surgeon.

In contrast to all the advantages just analyzed, it must be considered that robotic systems are costly, and not all orthopedic centers can implement them [36]. Furthermore, our study analyzes the application of robotic surgery in patients with minor deformities, but some studies, such as that by Yang et al. [37], highlight that rTKA also improved lower-limb coronal alignment, sagittal implant position, and early functional recovery for patients with severe varus/valgus deformity of the knee.

By conducting a critical and comprehensive review of the current scientific literature, we found some studies, such as that by Fozo et al. [8], which compare rTKA with manual TKA and show the superiority of conventional TKA over rTKA, as conventional TKA had lower operative time and tourniquet time. Additionally, the change in the hip–knee–ankle angle was superior in conventional TKA. Furthermore, as highlighted by the study of Hua and Salcedo [36], which analyzes the cost-effectiveness of these technologies, despite high robotic purchase costs, rTKA is likely to be cost-effective relative to TKA in the Medicare population with knee OA in high-volume hospitals by lowering revision rates and decreasing post-acute care costs.

### 4.4. Study Limitations and Future Directions

In this retrospective study, the generalizability of the findings may be limited due to several factors. The use of existing data introduces potential biases in patient selection and complicates the establishment of causality. Additionally, the specific patient population and surgical techniques examined may not accurately reflect broader demographics or various clinical settings, which could impact the applicability of the results. The relatively short follow-up period of the study may also fail to capture long-term outcomes and complications, further restricting the generalizability of the findings. To address these limitations, future prospective studies with larger sample sizes and extended follow-up are crucial. These studies should focus on key areas such as the long-term durability and performance of rTKA implants, their cost-effectiveness compared to traditional methods, and their impact on patient quality of life. The results do not provide detailed data on clinical outcomes, such as pain scores, patient satisfaction, functional improvement, or complication rates between the two groups. Additionally, there is a clear disparity in sample sizes, as the non-robotic group consisted of 141 patients compared with 247 patients in the robotic group.

By exploring these aspects, future research can provide more thorough insights into the efficacy and safety of robotic-assisted techniques, thereby improving clinical decision making and patient outcomes. Furthermore, it will be interesting to evaluate the impact of different types of rehabilitation, especially in conjunction with robotic surgery [13,38]. A shift towards prospective research is essential for gaining a comprehensive understanding of the role of robotics in modern orthopedic surgery.

## 5. Conclusions

Robotic planning in knee arthroplasty surgeries with minor deformities may be a useful tool to optimize alignment and implant fit. Preoperative imaging and robotic systems enable precise mapping of anatomical structures, allowing for customized implant positioning and alignment. Moreover, robotic technology allows for a reduced level of constraint in the intraoperative choice between Posterior-Stabilized and Constrained Posterior-Stabilized liners compared with an imageless navigated procedure. Future studies should focus on long-term outcomes of robotic-assisted knee arthroplasty, including patient satisfaction, implant longevity, and complication rates.

## Figures and Tables

**Table 1:** Demographic distribution.

**Gender**	110 (36.7%) Male	190 (63.3%) Female
**Age**	72.7 ± 8.9 years	
**Side**	150 Right	150 Left

**Table 2:** Types of femoral components.

Femoral Size	Number (%)
3	1 (0.3%)
4	8 (2.7%)
5	37 (12.3%)
6	49 (16.3%)
7	53 (17.7%)
8	47 (15.7%)
9	48 (16%)
10	25 (8.3%)
11	27 (9%)
12	5 (1.6%)

**Table 3:** Types of tibial components.

Tibial Size	Number (%)
C	7.3% (22)
D	31.7% (95)
E	25% (75)
F	20.3% (61)
G	13.7% (41)
H	2% (6)

**Table 4:** Types of inserts [PS: Posterior-Stabilized; MC: Medial Congruent; CPS: Constrained Posterior-Stabilized].

Inserts	Number (%)
PS insert	71.3% (214)
MC insert	17.7% (53)
CPS insert	11% (33)
Insert 10 mm	64.7% (194)
Insert 11 mm	23% (69)
Insert 12 mm	12.3% (37)

**Table 5:** Comparison of demographic characteristics between the group treated with iAssist (Zimmer Biomet, Warsaw, IN, USA) and Rosa Knee System (Zimmer Biomet, Warsaw, IN, USA).

	Rosa (*n* = 247)	iAssist (*n* = 141)	*p* Value
Age (years)	72.4 ± 8.4	70.7 ± 7.1	0.197
Male	87 (35.2%)	52 (36.9%)	0.372
Female	160 (64.8%)	89 (63.1%)	
Right	125 (50.6%)	67 (47.5%)	0.574
Left	122 (49.4%)	74 (52.5)	

**Table 6:** Comparison of insert type and relative height between the group treated with iAssist (Zimmer Biomet, Warsaw, IN, USA) and Rosa Knee System (Zimmer Biomet, Warsaw, IN, USA) [PS: Posterior-Stabilized; CPS: Constrained Posterior-Stabilized].

	Rosa (*n* = 247)	iAssist (*n* = 141)	*p* Value
PS	214 (86.7%)	111 (78.7%)	0.013
CPS	33 (13.3%)	30 (21.3%)	
10 mm	164 (66.4%)	86 (61%)	0.234
11 mm	51 (20.6%)	34 (24.1%)	
12 mm	32 (13%)	21 (14.9)	

## Data Availability

The data presented in this study are available on request from the corresponding author (privacy).
